# Supplementary material for: A set of multi-entry identification keys to African frugivorous flies (Diptera, Tephritidae)
Source: Zookeys. 2014 Jul 24;(428):97–108. doi: 10.3897/zookeys.428.7366 (PMC4143993; doi:10.3897/zookeys.428.7366)
Supplement: Supplementary material 6 — Key to Ceratitis [file zookeys-428-097-s006.zip › SF6_ZooKeys_key to Ceratitis/key/SF6_ZooKeys_key to Ceratitis/Media/Html/Ceratitis paracolae.htm]

Ceratitis (Pterandrus) paracolae, n


***Ceratitis*** **(*Pterandrus*) *paracolae* De Meyer & Freidberg**

Body
length: 5.34 (5.10-5.50) mm; wing length: 5.58 (5.20-5.80) mm.

Male

Head: Antenna yellowish orange. First flagellomere twice as
long as pedicel. Arista with moderately long rays; ventral rays shorter and
sparser than dorsal rays, especially basally. Frons convex; yellow; with short
scattered setulae of same color as frons. Frontal setae well developed. Face
yellowish white; gena dark, yellowish brown; mouthparts with apex partly
darkened but not completely black (as in lepida). Genal seta and setulae dark,
well developed, the latter with bushy appearance.

Thorax: Postpronotal
lobe white, without spot. Scutal pattern; ground color gray with silvery shine;
with streaks and darker markings but without distinct spots or clearly defined
stripes, except prescutellar white separate markings, almost reaching
dorsocentrals; presence of faint but indistinct yellow lateral stripes.
Scapular setae dark. Scutellum white, basally without spots, apically with three
separate black spots, extending to half-way. Anepisternum along ventral margin
brown; setulae pale except on ventral part, where black.

Legs:
Yellow except where otherwise noted; setation typical for subgenus, mainly
dark. Foreleg: coxa black; femur anteriorly dark brown except with patch
ventrodistally; posteriorly with dispersed dark setulae, no bush; ventral setae
dark; tibia anteriorly distinctly darkened along medial length. Midleg: femur
anteriorly brown with pale patches distally and anteroventrally; ventrally with
dark feathering along entire length except for interruption (8-9 setae wide) in
median part; tibia broadened; dark with pale patches, silvery shine when viewed
from certain angle; with black feathering dorsally along distal 0.8 and ventrally
along distal 0.66, anteriorly with few dispersed short dark setulae. Hindleg:
femur at apical 0.25 with longer setulae dorsally and ventrally, the latter
continued basally forming feathering.

Wing:
bands yellowish brown. Interruption between marginal and discal bands
near vein R_1_ clear and complete; cubital band free; medial band
absent; crossvein R-M proximal to middle of discal cell. Apex of vein R_1_
distal to level of crossvein R-M. Crossvein DM-Cu oblique anterobasally.

Abdomen:
Mostly yellow. Tergite 1 yellowish orange. Tergites 2 and 4 with pale gray
transverse band along� posterior half, band with weak silvery shine. Tergite 3
with well defined brown transverse band along� posterior 0.33 to half. Tergite
5 with yellowish brown patches along anterior part and weak silvery band
posteriorly, posterior margin also yellowish brown. Setation and banding
typical for subgenus. Male epandrium in lateral view with posterior lobe of
lateral surstylus short and stout, anterior lobe well pronounced.

Female

As
male except for the following characters: gena less dark, mouth parts not
darkened along apex. Anepisternum along ventral margin more yellowish brown.
Legs yellowish orange; without feathering, mainly with dark pilosity; forefemur
posteroventrally with dark pilosity. Crossvein DM-Cu oblique posterobasally.
Ovipositor shorter than preabdomen. Aculeus about seven times longer than wide;
tip with shoulder, and lateral margin slightly sinuous.

(Description
after De Meyer & Freidberg, 2006)
